# Equivalent property of a half-discrete Hilbert’s inequality with parameters

**DOI:** 10.1186/s13660-018-1926-1

**Published:** 2018-12-06

**Authors:** Zhenxiao Huang, Bicheng Yang

**Affiliations:** 1 Zhangjiang Preschool Education College, Suixi, P.R. China; 2 Department of Mathematics, Guangdong University of Education, Guangzhou, P.R. China

**Keywords:** 26D15, Weight function, Half-discrete Hilbert’s inequality, Equivalent form, Best possible constant factor

## Abstract

By using the weight functions and the idea of introducing parameters, a half-discrete Hilbert inequality with a nonhomogeneous kernel and its equivalent form are given. The equivalent statements of the constant factor are best possible related to parameters, and some particular cases are considered. The cases of a homogeneous kernel are also deduced.

## Introduction

If $0 < \sum_{m = 1}^{\infty } a_{m}^{2} < \infty ,0 < \sum_{n = 1}^{\infty } b_{n}^{2} < \infty $, then we have the following discrete Hilbert inequality with the best possible constant factor *π* [1]:

1 $$ \sum_{m = 1}^{\infty } \sum _{n = 1}^{\infty } \frac{a_{m}b_{n}}{m + n} < \pi \Biggl(\sum _{m = 1}^{\infty } a_{m}^{2} \sum_{n = 1}^{\infty } b_{n}^{2} \Biggr)^{1/2}. $$

Assuming that $0 < \int _{0}^{\infty } f^{2}(x)\,dx < \infty $, $0 < \int _{0}^{\infty } g^{2}(y)\,dy < \infty $, we still have the following Hilbert integral inequality [1]:

2 $$ \int _{0}^{\infty } \int _{0}^{\infty } \frac{f(x)g(y)}{x + y}\,dx\,dy < \pi \biggl( \int _{0}^{\infty } f^{2}(x)\,dx \int _{0}^{\infty } g^{2}(y)\,dy \biggr)^{1/2}, $$

where the constant factor *π* is the best possible. Inequalities (1) and (2) are important in analysis and its applications (cf. [2–13]).

We still have the following half-discrete Hilbert-type inequalities (cf. [1], Theorem 351): If $K(x)(x > 0)$ is decreasing, $p > 1$, $\frac{1}{p} + \frac{1}{q} = 1$, $0 < \phi (s) = \int _{0}^{\infty } K(x)x^{s - 1}\,dx <\infty $, then

3 $$\begin{aligned}& \int _{0}^{\infty } x^{p - 2} \Biggl(\sum _{n = 1}^{\infty } K(nx)a_{n} \Biggr)^{p} \,dx < \phi ^{p} \biggl(\frac{1}{q} \biggr)\sum _{n = 1}^{\infty } a_{n}^{p}, \end{aligned}$$

4 $$\begin{aligned}& \sum_{n = 1}^{\infty } n^{p - 2} \biggl( \int _{0}^{\infty } K (nx)f(x)\,dx \biggr)^{p} < \phi ^{p} \biggl(\frac{1}{q} \biggr) \int _{0}^{\infty } f^{p} (x)\,dx. \end{aligned}$$

In recent years, some new extensions of (3) and (4) were provided by [14–19].

In 2016, Hong [20, 21] also considered some equivalent statements of the extensions of (1) and (2) with a few parameters. For the following work we refer to [22–24].

In this paper, following [20], by the use of the weight functions and the idea of introducing parameters, a half-discrete Hilbert inequality with the nonhomogeneous kernel and its equivalent form are given. The equivalent statements of the constant factor are best possible related to parameters, and some particular cases are considered. The cases of a homogeneous kernel are also deduced.

## Some lemmas

In what follows, we assume that $p > 1$, $\frac{1}{p} + \frac{1}{q} =1$, $\lambda > 0$, *σ*, $\sigma _{1} \le 1$, $\sigma ,\sigma _{1} \in (0,\lambda ),f(x)$ is a nonnegative measurable function in $\mathbb{R}_{ +} = (0,\infty )$, $a_{n} \ge 0$ ($n \in \mathbb{N} = \{ 1,2, \ldots \} $), such that

$$ 0 < \int _{0}^{\infty } x^{p[1 - (\frac{\sigma }{p} + \frac{\sigma _{1}}{q})] - 1}f^{p}(x)\,dx< \infty ,\qquad 0 < \sum_{n = 1}^{\infty } n^{q[1 - (\frac{\sigma }{p} + \frac{\sigma _{1}}{q})] - 1} a_{n}^{q} < \infty . $$

### Lemma 1

*Define the following weight functions*:

5 $$\begin{aligned}& \omega _{\sigma } (\sigma _{1},n): = n^{\sigma } \int _{0}^{\infty } \frac{1}{(1 + xn)^{\lambda }} x^{\sigma _{1} - 1}\,dx \quad (n \in \mathbb{N}), \end{aligned}$$

6 $$\begin{aligned}& \varpi _{\sigma _{1}}(\sigma ,x): = x^{\sigma _{1}}\sum _{n = 1}^{\infty } \frac{1}{(1 + xn)^{\lambda }} n^{\sigma - 1} \quad (x \in \mathbb{R}_{ +} ). \end{aligned}$$

*We have the following equality and inequalities*:

7 $$\begin{aligned}& \omega _{\sigma } (\sigma _{1},n) = B(\sigma _{1}, \lambda - \sigma _{1})n^{\sigma - \sigma _{1}}\quad (n \in \mathbb{N}), \end{aligned}$$

8 $$\begin{aligned}& \biggl(B(\sigma ,\lambda - \sigma ) - \frac{x^{\sigma }}{\sigma } \biggr)x^{\sigma _{1} - \sigma } < \varpi _{\sigma _{1}}(\sigma ,x) < B(\sigma ,\lambda - \sigma ) \cdot x^{\sigma _{1} - \sigma } \quad (x \in \mathbb{R}_{ +} ), \end{aligned}$$

*where* $B(u,v): = \int _{0}^{\infty } \frac{t^{u - 1}}{(1 + t)^{u + v}}\,dt$ ($u,v> 0$) *is the Beta function*.

### Proof

Setting $u = xn$, we have

$$\begin{aligned} \omega _{\sigma } (\sigma _{1},n) &= n^{\sigma } \int _{0}^{\infty } \frac{1}{(1 + u)^{\lambda }} \biggl( \frac{u}{n} \biggr)^{\sigma _{1} - 1} \frac{1}{n}\,du \\ &= n^{\sigma - \sigma _{1}} \int _{0}^{\infty } \frac{u^{\sigma _{1} - 1}}{(1 + u)^{\lambda }}\,du \\ &= B(\sigma _{1},\lambda - \sigma _{1})n^{\sigma -\sigma _{1}}, \end{aligned}$$

and then (7) follows. In view of the decreasing property, we find

$$\begin{aligned} \varpi _{\sigma _{1}}(\sigma ,x) &< x^{\sigma _{1}} \int _{0}^{\infty } \frac{t^{\sigma - 1}}{(1 + xt)^{\lambda }}\,dt \\ &= x^{\sigma _{1} - \sigma } \int _{0}^{\infty } \frac{u^{\sigma - 1}}{(1 + u)^{\lambda }}\,du \\ &= x^{\sigma _{1} - \sigma } B(\sigma ,\lambda - \sigma ), \\ \varpi _{\sigma _{1}}(\sigma ,x)&> x^{\sigma _{1}} \int _{1}^{\infty } \frac{t^{\sigma - 1}}{(1 + xt)^{\lambda }}\,dt \\ &= x^{\sigma _{1} - \sigma } \int _{x}^{\infty } \frac{u^{\sigma - 1}}{(1 + u)^{\lambda }}\,du \\ &= x^{\sigma _{1} - \sigma } \biggl[ \int _{0}^{\infty } \frac{u^{\sigma - 1}\,du}{(1 + u)^{\lambda }} - \int _{0}^{x} \frac{u^{\sigma - 1}\,du}{(1 + u)^{\lambda }} \biggr] \ge x^{\sigma _{1} - \sigma } \biggl(B(\sigma ,\lambda - \sigma ) - \int _{0}^{x} u^{\sigma - 1}\,du \biggr) \\ &= x^{\sigma _{1} - \sigma } \biggl(B(\sigma ,\lambda - \sigma ) - \frac{x^{\sigma }}{\sigma } \biggr). \end{aligned}$$

Hence, (8) follows. □

### Lemma 2

*Setting* $k_{\lambda } (\eta ): = B(\eta ,\lambda - \eta )(\eta = \sigma ,\sigma _{1})$, *we have the following inequality*:

9 $$\begin{aligned} I&: = \int _{0}^{\infty } \sum_{n = 1}^{\infty } \frac{a_{n}}{(1 + xn)^{\lambda }} f(x)\,dx \\ &= \sum_{n = 1}^{\infty } \int _{0}^{\infty } \frac{a_{n}}{(1 + xn)^{\lambda }} f(x)\,dx \\ &< k_{\lambda }^{\frac{1}{p}}(\sigma )k_{\lambda }^{\frac{1}{q}}( \sigma _{1}) \biggl\{ \int _{0}^{\infty } x^{p[1 - (\frac{\sigma }{p} + \frac{\sigma _{1}}{q})] - 1}f^{p}(x)\,dx \biggr\} ^{\frac{1}{p}} \Biggl\{ \sum_{n = 1}^{\infty } n^{q[1 - (\frac{\sigma }{p} + \frac{\sigma _{1}}{q})] - 1} a_{n}^{q} \Biggr\} ^{\frac{1}{q}}. \end{aligned}$$

### Proof

By Hölder’s inequality (cf. [25]), we have

$$\begin{aligned} I &= \int _{0}^{\infty } \sum_{n = 1}^{\infty } \frac{1}{(1 + xn)^{\lambda }} \biggl[\frac{x^{(1 - \sigma _{1})/q}}{n^{(1 - \sigma )/p}}f(x) \biggr] \biggl[ \frac{n^{(1 - \sigma )/p}}{x^{(1 - \sigma _{1})/q}}a_{n} \biggr]\,dx \\ &\le \Biggl[ \int _{0}^{\infty } \sum_{n = 1}^{\infty } \frac{1}{(1 + xn)^{\lambda }} \frac{x^{(1 - \sigma _{1})p/q}}{n^{1 - \sigma }} f^{p}(x)\,dx \Biggr]^{\frac{1}{p}} \Biggl[\sum_{n = 1}^{\infty } \int _{0}^{\infty } \frac{1}{(1 + xn)^{\lambda }} \frac{n^{(1 - \sigma )q/p}}{x^{1 - \sigma _{1}}} \,dx\,a_{n}^{q} \Biggr]^{\frac{1}{q}} \\ &= \biggl[\int _{0}^{\infty } \varpi _{\sigma _{1}}(\sigma ,x)x^{p(1 - \sigma _{1}) - 1}f^{p}(x)\,dx \biggr]^{\frac{1}{p}} \Biggl[\sum _{n = 1}^{\infty } \omega _{\sigma } (\sigma _{1},n) n^{q(1 - \sigma ) - 1}a_{n}^{q}\Biggr]^{\frac{1}{q}}. \end{aligned}$$

Then, by (7) and (8), we have (9). □

By (9), for $\sigma _{1} = \sigma $, we find $0 < \int _{0}^{\infty } x^{p(1 - \sigma ) - 1}f^{p}(x)\,dx < \infty $, $0 < \sum_{n = 1}^{\infty } n^{q(1 - \sigma ) - 1} a_{n}^{q} < \infty $, and

10 $$ \int _{0}^{\infty } \sum_{n = 1}^{\infty } \frac{a_{n}}{(1 + xn)^{\lambda }} f(x)\,dx< k_{\lambda } (\sigma ) \biggl[ \int _{0}^{\infty } x^{p(1 - \sigma ) - 1}f^{p}(x)\,dx \biggr]^{\frac{1}{p}} \Biggl[\sum_{n = 1}^{\infty } n^{q(1 - \sigma ) - 1} a_{n}^{q} \Biggr]^{\frac{1}{q}}. $$

### Lemma 3

*The constant factor* $k_{\lambda } (\sigma ) = B(\sigma ,\lambda - \sigma )$ *in* (10) *is the best possible*.

### Proof

For $0 < \varepsilon < q\sigma $, we set

$$ \tilde{a}_{n} = n^{\sigma - \frac{\varepsilon }{q} - 1}\quad (n \in \mathbb{N}),\qquad \tilde{f}(x) = \textstyle\begin{cases} \sigma + \frac{\varepsilon }{p} - 1,&0 < x \le 1, \\ 0,&x > 1. \end{cases} $$

If there exists a constant $M \le k_{\lambda } (\sigma )$, such that (10) is valid when replacing $k_{\lambda } (\sigma )$ by *M*, then, for $a_{n} = \tilde{a}_{n}$, $f = \tilde{f}$, we have

$$ \tilde{I}: = \int _{0}^{\infty } \sum_{n = 1}^{\infty } \frac{\tilde{a}_{n}}{(1 + xn)^{\lambda }} \tilde{f}(x)\,dx< M \biggl[ \int _{0}^{\infty } x^{p(1 - \sigma ) - 1}\tilde{f}^{p}(x) \,dx \biggr]^{\frac{1}{p}} \Biggl[\sum_{n = 1}^{\infty } n^{q(1 - \sigma ) - 1} \tilde{a}_{n}^{q} \Biggr]^{\frac{1}{q}}. $$

We obtain

$$\begin{aligned} \tilde{I} &< M\biggl[ \int _{0}^{1} x^{p(1 - \sigma ) - 1}x^{p(\sigma + \frac{\varepsilon }{p} - 1)}\,dx \biggr]^{\frac{1}{p}}\Biggl[\sum_{n = 1}^{\infty } n^{q(1 - \sigma ) - 1} n^{q(\sigma - \frac{\varepsilon }{q} - 1)}\Biggr]^{\frac{1}{q}} \\ &= M\biggl( \int _{0}^{1} x^{\varepsilon - 1}\,dx \biggr)^{\frac{1}{p}}\Biggl(1 + \sum_{n = 2}^{\infty } n^{ - \varepsilon - 1} \Biggr)^{\frac{1}{q}} \\ &< M\biggl( \int _{0}^{1} x^{\varepsilon - 1}\,dx \biggr)^{\frac{1}{p}}\biggl(1 + \int _{1}^{\infty } x^{ - \varepsilon - 1}\,dx \biggr)^{\frac{1}{q}} \\ &= \frac{M}{\varepsilon } (\varepsilon + 1)^{\frac{1}{q}}. \end{aligned}$$

In view of (8), we find

$$\begin{aligned} \tilde{I} &= \int _{0}^{1} x^{\varepsilon - 1} \Biggl[x^{(\sigma - \frac{\varepsilon }{q})} \sum_{n = 1}^{\infty } \frac{1}{(1 + xn)^{\lambda }} n^{(\sigma - \frac{\varepsilon }{q}) - 1} \Biggr]\,dx \\ &> \int _{0}^{1} x^{\varepsilon - 1} \biggl(B \biggl( \sigma - \frac{\varepsilon }{q},\lambda - \sigma + \frac{\varepsilon }{q} \biggr) - \frac{x^{\sigma - \frac{\varepsilon }{q}}}{\sigma - \frac{\varepsilon }{q}} \biggr)\,dx \\ & = \frac{1}{\varepsilon } B\biggl(\sigma - \frac{\varepsilon }{q},\lambda - \sigma + \frac{\varepsilon }{q}\biggr) - \frac{1}{\sigma - \frac{\varepsilon }{q}} \int _{0}^{1} x^{\sigma + \frac{\varepsilon }{p} - 1}\,dx \\ &= \frac{1}{\varepsilon } \biggl[B\biggl(\sigma - \frac{\varepsilon }{q},\lambda - \sigma + \frac{\varepsilon }{q}\biggr) - \frac{\varepsilon }{(\sigma - \frac{\varepsilon }{q})(\sigma + \frac{\varepsilon }{p})}\biggr]. \end{aligned}$$

Then we have

$$ B \biggl(\sigma - \frac{\varepsilon }{q},\lambda - \sigma + \frac{\varepsilon }{q} \biggr) - \frac{\varepsilon }{(\sigma - \frac{\varepsilon }{q})(\sigma + \frac{\varepsilon }{p})} < M(\varepsilon + 1)^{\frac{1}{q}}. $$

For $\varepsilon \to 0^{ +} $, in view of the continuous property of the Beta function, we find

$$\begin{aligned} B(\sigma ,\lambda - \sigma ) &= \lim_{\varepsilon \to 0^{ +}} \biggl[B\biggl( \sigma - \frac{\varepsilon }{q},\lambda - \sigma + \frac{\varepsilon }{q}\biggr) - \frac{\varepsilon }{(\sigma - \frac{\varepsilon }{q})(\sigma + \frac{\varepsilon }{p})}\biggr] \\ &\le \lim_{\varepsilon \to 0^{ +}} M(\varepsilon + 1)^{\frac{1}{q}} = M. \end{aligned}$$

Hence, $M = B(\sigma ,\lambda - \sigma )$ is the best possible constant factor of (10). □

Setting $\tilde{\sigma } = \frac{\sigma }{p} + \frac{\sigma _{1}}{q}$ ($\sigma ,\sigma _{1} \le 1,\sigma ,\sigma _{1} \in (0,\lambda )$), we may rewrite (9) as follows:

11 $$ I < k_{\lambda }^{\frac{1}{p}}(\sigma )k_{\lambda }^{\frac{1}{q}}( \sigma _{1}) \biggl[ \int _{0}^{\infty } x^{p(1 - \tilde{\sigma } ) - 1}f^{p}(x)\,dx \biggr]^{\frac{1}{p}} \Biggl[\sum_{n = 1}^{\infty } n^{q(1 - \tilde{\sigma } ) - 1} a_{n}^{q} \Biggr]^{\frac{1}{q}}. $$

The parameter *σ̃* in (11) also satisfies

12 $$\begin{aligned} 0 &< k_{\lambda } (\tilde{\sigma } ) = k_{\lambda } \biggl( \frac{\sigma }{p} + \frac{\sigma _{1}}{q} \biggr) \\ &= \int _{0}^{\infty } \frac{1}{(1 + u)^{\lambda }} \bigl(u^{\frac{\sigma - 1}{p}} \bigr) \bigl(u^{\frac{\sigma _{1} - 1}{q}} \bigr)\,du \\ &\le \biggl[ \int _{0}^{\infty } \frac{u^{\sigma - 1}}{(1 + u)^{\lambda }}\,du \biggr]^{\frac{1}{p}} \biggl[ \int _{0}^{\infty } \frac{u^{\sigma _{1} - 1}}{(1 + u)^{\lambda }}\,du \biggr]^{\frac{1}{q}} \\ &= k_{\lambda }^{\frac{1}{p}}(\sigma )k_{\lambda }^{\frac{1}{q}}(\sigma _{1}) \\ &< \infty , \end{aligned}$$

by Hölder’s inequality, and $\tilde{\sigma } \le \frac{1}{p} + \frac{1}{q} = 1$, $\tilde{\sigma } \in (0,\lambda )$, such that

$$ k_{\lambda } (\tilde{\sigma } ) - \frac{x^{\tilde{\sigma }}}{\tilde{\sigma }} < x^{\tilde{\sigma }} \sum _{n = 1}^{\infty } \frac{n^{\tilde{\sigma } - 1}}{(1 + xn)^{\lambda }} < k_{\lambda } (\tilde{\sigma } ). $$

### Lemma 4

*If the constant factor* $k_{\lambda }^{\frac{1}{p}}(\sigma )k_{\lambda }^{\frac{1}{q}}(\sigma _{1})$ *in* (11) *is the best possible*, *then we have* $\sigma _{1} = \sigma $.

### Proof

If the constant factor $k_{\lambda }^{\frac{1}{p}}(\sigma )k_{\lambda }^{\frac{1}{q}}(\sigma _{1})$ in (11) is the best possible, then, by (10), the unique best possible constant factor must be $k_{\lambda } (\tilde{\sigma } )$, namely, $k_{\lambda } (\tilde{\sigma } ) = k_{\lambda }^{\frac{1}{p}}(\sigma )k_{\lambda }^{\frac{1}{q}}(\sigma _{1})$. Since the condition of (12) keeps the form of equality is that there exist constants *A* and *B*, such that they are not all zero and $Au^{\sigma - 1} =Bu^{\sigma _{1} - 1}$ a.e. in $\mathbb{R}_{ +}$. Assuming that $A \ne0 $, it follows that $u^{\sigma - \sigma _{1}} = \frac{B}{A}$ a.e. in $\mathbb{R}_{ +} $, and then $\sigma - \sigma _{1} = 0$, namely, $\sigma _{1} = \sigma$. □

## Main results and some corollaries

### Theorem 1

*Inequality* (9) *is equivalent to the following inequalities*:

13 $$\begin{aligned} J_{1}&: = \Biggl\{ \sum_{n = 1}^{\infty } n^{p(\frac{\sigma }{p} + \frac{\sigma _{1}}{q}) - 1} \biggl[ \int _{0}^{\infty } \frac{f(x)}{(1 + xn)^{\lambda }}\,dx \biggr]^{p} \Biggr\} ^{\frac{1}{p}} \\ &< k_{\lambda }^{\frac{1}{p}}(\sigma )k_{\lambda }^{\frac{1}{q}}( \sigma _{1}) \biggl\{ \int _{0}^{\infty } x^{p[1 - (\frac{\sigma }{p} + \frac{\sigma _{1}}{q})] - 1}f^{p}(x)\,dx \biggr\} ^{\frac{1}{p}}, \end{aligned}$$

14 $$\begin{aligned} J_{2}&: = \Biggl\{ \int _{0}^{\infty } x^{q(\frac{\sigma }{p} + \frac{\sigma _{1}}{q}) - 1} \Biggl[\sum _{n = 1}^{\infty } \frac{a_{n}}{(1 + xn)^{\lambda }} \Biggr]^{q} \,dx \Biggr\} ^{\frac{1}{q}} \\ &< k_{\lambda }^{\frac{1}{p}}(\sigma )k_{\lambda }^{\frac{1}{q}}( \sigma _{1}) \Biggl\{ \sum_{n = 1}^{\infty } n^{q[1 - (\frac{\sigma }{p} + \frac{\sigma _{1}}{q})] - 1} a_{n}^{q} \Biggr\} ^{\frac{1}{q}}. \end{aligned}$$

*If the constant factor in* (9) *is the best possible*, *then so is the constant factor in* (13) *and* (14).

### Proof

Suppose that (13) (or (14)) is valid. By Hölder’s inequality, we have

15 $$\begin{aligned} I &= \sum_{n = 1}^{\infty } \biggl[n^{\frac{ - 1}{p} + (\frac{\sigma }{p} + \frac{\sigma _{1}}{q})} \int _{0}^{\infty } \frac{f(x)}{(1 + xn)^{\lambda }}\,dx \biggr] \bigl[n^{\frac{1}{p} - (\frac{\sigma }{p} + \frac{\sigma _{1}}{q})}a_{n} \bigr] \\ &\le J_{1} \Biggl\{ \sum_{n = 1}^{\infty } n^{q[1 - (\frac{\sigma }{p} + \frac{\sigma _{1}}{q})] - 1} a_{n}^{q} \Biggr\} ^{\frac{1}{q}}, \end{aligned}$$

16 $$\begin{aligned} I &= \int _{0}^{\infty } \bigl[x^{\frac{1}{q} - (\frac{\sigma }{p} + \frac{\sigma _{1}}{q})}f(x) \bigr] \Biggl[x^{\frac{ - 1}{q} + (\frac{\sigma }{p} + \frac{\sigma _{1}}{q})}\sum_{n = 1}^{\infty } \frac{1}{(1 + xn)^{\lambda }} a_{n} \Biggr]\,dx \\ &\le \biggl\{ \int _{0}^{\infty } x^{p[1 - (\frac{\sigma }{p} + \frac{\sigma _{1}}{q})] - 1}f^{p}(x)\,dx \biggr\} ^{\frac{1}{p}}J_{2}. \end{aligned}$$

Then, by (13) (or (14)), we have (9). On the other hand, assuming (9) is valid, we set

$$ a_{n}: = n^{p(\frac{\sigma }{p} + \frac{\sigma _{1}}{q}) - 1} \biggl[ \int _{0}^{\infty } \frac{f(x)}{(1 + xn)^{\lambda }}\,dx \biggr]^{p - 1}\quad (n \in \mathbb{N}). $$

If $J_{1} = 0$, then (13) is naturally valid; if $J_{1} = \infty $, then it is impossible that it makes (13) valid. Suppose that $0 < J_{1} < \infty $. By (9) we have

$$\begin{aligned}& \sum_{n = 1}^{\infty } n^{q[1 - (\frac{\sigma }{p} + \frac{\sigma _{1}}{q})] - 1}a_{n}^{q} \\& \quad = J_{1}^{p} = I < k_{\lambda }^{\frac{1}{p}}( \sigma )k_{\lambda }^{\frac{1}{q}}(\sigma _{1}) \biggl\{ \int _{0}^{\infty } x^{p[1 - (\frac{\sigma }{p} + \frac{\sigma _{1}}{q})] - 1}f^{p}(x)\,dx \biggr\} ^{\frac{1}{p}} \Biggl\{ \sum_{n = 1}^{\infty } n^{q[1 - (\frac{\sigma }{p} + \frac{\sigma _{1}}{q})] - 1} a_{n}^{q} \Biggr\} ^{\frac{1}{q}}, \\& \Biggl\{ \sum_{n = 1}^{\infty } n^{q[1 - (\frac{\sigma }{p} + \frac{\sigma _{1}}{q})] - 1} a_{n}^{q} \Biggr\} ^{\frac{1}{p}} = J_{1} < k_{\lambda }^{\frac{1}{p}}(\sigma )k_{\lambda }^{\frac{1}{q}}(\sigma _{1}) \biggl\{ \int _{0}^{\infty } x^{p[1 - (\frac{\sigma }{p} + \frac{\sigma _{1}}{q})] - 1}f^{p}(x)\,dx \biggr\} ^{\frac{1}{p}}, \end{aligned}$$

namely, (13) follows.

In the same way, assuming (9) is valid, we set

$$ f(x): = x^{q(\frac{\sigma }{p} + \frac{\sigma _{1}}{q}) - 1} \Biggl[\sum_{n = 1}^{\infty } \frac{1}{(1 + xn)^{\lambda }} a_{n} \Biggr]^{q - 1}\quad (x \in \mathbb{R}). $$

If $J_{2} = 0$, then (14) is naturally valid; if $J_{2} = \infty $, then it is impossible that makes (14) valid. Suppose that $0 < J_{2} < \infty $. By (9) we have

$$\begin{aligned}& \int _{0}^{\infty } x^{p[1 - (\frac{\sigma }{p} + \frac{\sigma _{1}}{q})] - 1}f^{p}(x)\,dx \\& \quad =J_{2}^{q} = I< k_{\lambda }^{\frac{1}{p}}( \sigma )k_{\lambda }^{\frac{1}{q}}(\sigma _{1}) \biggl\{ \int _{0}^{\infty } x^{p[1 - (\frac{\sigma }{p} + \frac{\sigma _{1}}{q})] - 1}f^{p}(x)\,dx \biggr\} ^{\frac{1}{p}} \Biggl\{ \sum_{n = 1}^{\infty } n^{q[1 - (\frac{\sigma }{p} + \frac{\sigma _{1}}{q})] - 1} a_{n}^{q} \Biggr\} ^{\frac{1}{q}}, \\& \biggl\{ \int _{0}^{\infty } x^{p[1 - (\frac{\sigma }{p} + \frac{\sigma _{1}}{q})] - 1}f^{p}(x)\,dx \biggr\} ^{\frac{1}{q}} = J_{2} < k_{\lambda }^{\frac{1}{p}}( \sigma )k_{\lambda }^{\frac{1}{q}}(\sigma _{1}) \Biggl\{ \sum _{n = 1}^{\infty } n^{q[1 - (\frac{\sigma }{p} + \frac{\sigma _{1}}{q})] - 1} a_{n}^{q} \Biggr\} ^{\frac{1}{q}}, \end{aligned}$$

namely, (14) follows. Hence, inequalities (9), (13) and (14) are equivalent.

If the constant factor in (9) is the best possible, then so is constant factor in (13) (or (14)). Otherwise, by (15) (or (16)), we would reach the contradiction that the constant factor in (9) is not the best possible. □

### Theorem 2

*The statements* (i), (ii), (iii) *and* (iv) *are equivalent*:

(i) $k_{\lambda }^{\frac{1}{p}}(\sigma )k_{\lambda }^{\frac{1}{q}}(\sigma _{1})$ *is independent of* $p,q$;
(ii) $k_{\lambda }^{\frac{1}{p}}(\sigma)k_{\lambda }^{\frac{1}{q}}(\sigma _{1})$ *is expressed as a single integral*;
(iii) $k_{\lambda }^{\frac{1}{p}}(\sigma)k_{\lambda }^{\frac{1}{q}}(\sigma _{1})$ *in* (9) *is the best possible constant*;
(iv) $\sigma _{1} = \sigma$.

*If the statement* (iv) *follows*, *then we have the following equivalent inequalities with the best possible constant factor* $B(\sigma ,\lambda - \sigma )$:

17 $$\begin{aligned}& \int _{0}^{\infty } \sum_{n = 1}^{\infty } \frac{a_{n}}{(1 + xn)^{\lambda }} f(x)\,dx \\& \quad < B(\sigma ,\lambda - \sigma ) \biggl[ \int _{0}^{\infty } x^{p(1 - \sigma ) - 1}f^{p}(x)\,dx \biggr]^{\frac{1}{p}} \Biggl[\sum_{n = 1}^{\infty } n^{q(1 - \sigma ) - 1} a_{n}^{q} \Biggr]^{\frac{1}{q}}. \end{aligned}$$

18 $$\begin{aligned}& \Biggl[\sum_{n = 1}^{\infty } n^{p\sigma - 1} \biggl( \int _{0}^{\infty } \frac{f(x)}{(1 + xn)^{\lambda }}\,dx \biggr)^{p} \Biggr]^{\frac{1}{p}} \\& \quad < B(\sigma ,\lambda - \sigma ) \biggl[ \int _{0}^{\infty } x^{p(1 - \sigma ) - 1}f^{p}(x)\,dx \biggr]^{\frac{1}{p}}, \end{aligned}$$

19 $$\begin{aligned}& \Biggl[ \int _{0}^{\infty } x^{q\sigma - 1} \Biggl(\sum _{n = 1}^{\infty } \frac{a_{n}}{(1 + xn)^{\lambda }} \Biggr)^{q} \,dx \Biggr]^{\frac{1}{q}} \\& \quad < B(\sigma ,\lambda - \sigma ) \Biggl[\sum_{n = 1}^{\infty } n^{q(1 - \sigma ) - 1} a_{n}^{q} \Biggr]^{\frac{1}{q}}. \end{aligned}$$

### Proof

(i)⇒(ii). By (i) we have

$$ k_{\lambda }^{\frac{1}{p}}(\sigma )k_{\lambda }^{\frac{1}{q}}(\sigma _{1}) = \lim_{p \to 1^{ +}} k_{\lambda }^{\frac{1}{p}}( \sigma )k_{\lambda }^{\frac{1}{q}}(\sigma _{1}) = k_{\lambda } (\sigma ), $$

namely, $k_{\lambda }^{\frac{1}{p}}(\sigma )k_{\lambda }^{\frac{1}{q}}(\sigma _{1})$ is expressed as a single integral.

(ii)⇒(iv). In (12), if $k_{\lambda }^{\frac{1}{p}}(\sigma )k_{\lambda }^{\frac{1}{q}}(\sigma _{1})$ is expressed as a single integral $k_{\lambda } (\frac{\sigma }{p} + \frac{\sigma _{1}}{q})$, then (12) keeps the form of equality. In view of the proof of Lemma 4, if and only if $\sigma _{1} = \sigma $, (12) keeps the form of equality.

(iv)⇒(i). If $\sigma _{1} = \sigma $, then $k_{\lambda }^{\frac{1}{p}}(\sigma )k_{\lambda }^{\frac{1}{q}}(\sigma _{1}) = k_{\lambda } (\sigma )$, which is independent of $p,q$. Hence, we have (i) ↔ (ii) ⇔ (iv).

(iii)⇒(iv). By Lemma 4, we have $\sigma _{1} = \sigma $. (iv)⇒(iii). By Lemma 3, $k_{\lambda }^{\frac{1}{p}}(\sigma )k_{\lambda }^{\frac{1}{q}}(\sigma _{1}) = k_{\lambda } (\sigma )$ in (9) (for $\sigma _{1} = \sigma $) is the best possible constant. Therefore, we have (iii) ⇔ (iv).

Hence, the statements (i), (ii), (iii) and (iv) are equivalent. □

Replacing *x* by $\frac{1}{x}$, and then $x^{\lambda - 2}f(\frac{1}{x})$ by $f(x)$ in Theorem 1, setting $\sigma _{1} = \lambda - \mu $, we have the following.

### Corollary 1

*The following inequalities with the homogeneous kernel are equivalent*:

20 $$\begin{aligned} &\int _{0}^{\infty } \sum_{n = 1}^{\infty } \frac{a_{n}}{(x + n)^{\lambda }} f(x)\,dx \\ &\quad < k_{\lambda }^{\frac{1}{p}}(\sigma )k_{\lambda }^{\frac{1}{q}}( \lambda - \mu ) \biggl\{ \int _{0}^{\infty } x^{p[1 - (\frac{\lambda - \sigma }{p} + \frac{\mu }{q})] - 1}f^{p}(x)\,dx \biggr\} ^{\frac{1}{p}} \Biggl\{ \sum_{n = 1}^{\infty } n^{q[1 - (\frac{\sigma }{p} + \frac{\lambda - \mu }{q})] - 1} a_{n}^{q} \Biggr\} ^{\frac{1}{q}}. \end{aligned}$$

21 $$\begin{aligned} &\Biggl\{ \sum_{n = 1}^{\infty } n^{p(\frac{\sigma }{p} + \frac{\lambda - \mu }{q}) - 1} \biggl[ \int _{0}^{\infty } \frac{f(x)}{(x + n)^{\lambda }}\,dx \biggr]^{p} \Biggr\} ^{\frac{1}{p}} \\ &\quad < k_{\lambda }^{\frac{1}{p}}(\sigma )k_{\lambda }^{\frac{1}{q}}( \lambda - \mu ) \biggl\{ \int _{0}^{\infty } x^{p[1 - (\frac{\lambda - \sigma }{p} + \frac{\mu }{q})] - 1}f^{p}(x)\,dx \biggr\} ^{\frac{1}{p}}, \end{aligned}$$

22 $$\begin{aligned} &\Biggl\{ \int _{0}^{\infty } x^{q(\frac{\lambda - \sigma }{p} + \frac{\mu }{q}) - 1} \Biggl[\sum _{n = 1}^{\infty } \frac{a_{n}}{(x + n)^{\lambda }} \Biggr]^{q} \,dx \Biggr\} ^{\frac{1}{q}} \\ &\quad < k_{\lambda }^{\frac{1}{p}}(\sigma )k_{\lambda }^{\frac{1}{q}}( \lambda - \mu ) \Biggl\{ \sum_{n = 1}^{\infty } n^{q[1 - (\frac{\sigma }{p} + \frac{\lambda - \mu }{q})] - 1} a_{n}^{q} \Biggr\} ^{\frac{1}{q}}. \end{aligned}$$

If the constant factor in (20) is the best possible, then so is the constant factor in (21) and (22).

### Corollary 2

*The statements* (I), (II), (III) *and* (IV) *are equivalent*:

(I) $k_{\lambda }^{\frac{1}{p}}(\sigma )k_{\lambda }^{\frac{1}{q}}(\lambda - \mu )$ *is independent of* $p,q$;
(II) $k_{\lambda }^{\frac{1}{p}}(\sigma )k_{\lambda }^{\frac{1}{q}}(\lambda - \mu )$ *is expressed as a single integral*;
(III) $k_{\lambda }^{\frac{1}{p}}(\sigma )k_{\lambda }^{\frac{1}{q}}(\lambda - \mu )$ *in* (20) *is the best possible constant*;
(IV) $\mu + \sigma = \lambda$.

*If the statement* (IV) *follows*, *then we have the following equivalent inequalities with the best possible constant factor* $B(\mu ,\sigma )$:

23 $$ \int _{0}^{\infty } \sum_{n = 1}^{\infty } \frac{a_{n}}{(x + n)^{\lambda }} f(x)\,dx< B(\mu ,\sigma ) \biggl[ \int _{0}^{\infty } x^{p(1 - \mu ) - 1}f^{p}(x)\,dx \biggr]^{\frac{1}{p}} \Biggl[\sum_{n = 1}^{\infty } n^{q(1 - \sigma ) - 1} a_{n}^{q} \Biggr]^{\frac{1}{q}}. $$

24 $$ \Biggl\{ \sum_{n = 1}^{\infty } n^{p\sigma - 1} \biggl[ \int _{0}^{\infty } \frac{f(x)}{(x + n)^{\lambda }}\,dx \biggr]^{p} \Biggr\} ^{\frac{1}{p}}< B(\mu ,\sigma ) \biggl[ \int _{0}^{\infty } x^{p(1 - \sigma ) - 1}f^{p}(x)\,dx \biggr]^{\frac{1}{p}}, $$

25 $$ \Biggl\{ \int _{0}^{\infty } x^{q\mu - 1} \Biggl[\sum _{n = 1}^{\infty } \frac{a_{n}}{(x + n)^{\lambda }} \Biggr]^{q} \,dx \Biggr\} ^{\frac{1}{q}}< B(\mu ,\sigma ) \Biggl[\sum _{n = 1}^{\infty } n^{q(1 - \sigma ) - 1} a_{n}^{q} \Biggr]^{\frac{1}{q}}. $$

### Remark 1

(i) For $\sigma = \frac{1}{p}( < \lambda )$ in (17), (18) and (19), we have the following equivalent inequalities with the nonhomogeneous kernel and the best possible constant factor $B(\frac{1}{p},\lambda - \frac{1}{p})$:

26 $$ \int _{0}^{\infty } \sum_{n = 1}^{\infty } \frac{a_{n}}{(1 + xn)^{\lambda }} f(x)\,dx< B \biggl(\frac{1}{p},\lambda - \frac{1}{p} \biggr) \biggl( \int _{0}^{\infty } x^{p - 2}f^{p}(x)\,dx \biggr)^{\frac{1}{p}} \Biggl(\sum_{n = 1}^{\infty } a_{n}^{q} \Biggr)^{\frac{1}{q}}. $$

27 $$ \Biggl\{ \sum_{n = 1}^{\infty } \biggl[ \int _{0}^{\infty } \frac{f(x)}{(1 + xn)^{\lambda }}\,dx \biggr]^{p} \Biggr\} ^{\frac{1}{p}}< B \biggl(\frac{1}{p},\lambda - \frac{1}{p} \biggr) \biggl( \int _{0}^{\infty } x^{p - 2}f^{p}(x)\,dx \biggr)^{\frac{1}{p}}, $$

28 $$ \Biggl\{ \int _{0}^{\infty } x^{q - 2} \Biggl[\sum _{n = 1}^{\infty } \frac{a_{n}}{(1 + xn)^{\lambda }} \Biggr]^{q} \,dx \Biggr\} ^{\frac{1}{q}}< B \biggl(\frac{1}{p},\lambda - \frac{1}{p} \biggr) \Biggl(\sum_{n = 1}^{\infty } a_{n}^{q} \Biggr)^{\frac{1}{q}}. $$

(ii) For $\sigma = \frac{1}{q}( < \lambda )$ in (17), (18) and (19), we have the following equivalent inequalities with the best possible constant factor $B(\frac{1}{q},\lambda - \frac{1}{q})$:

29 $$ \int _{0}^{\infty } \sum_{n = 1}^{\infty } \frac{a_{n}}{(1 + xn)^{\lambda }} f(x)\,dx< B \biggl(\frac{1}{q},\lambda - \frac{1}{q} \biggr) \biggl( \int _{0}^{\infty } f^{p}(x)\,dx \biggr)^{\frac{1}{p}} \Biggl(\sum_{n = 1}^{\infty } n^{q - 2}a_{n}^{q} \Biggr)^{\frac{1}{q}}. $$

30 $$ \Biggl\{ \sum_{n = 1}^{\infty } n^{p - 2} \biggl[ \int _{0}^{\infty } \frac{f(x)}{(1 + xn)^{\lambda }}\,dx \biggr]^{p} \Biggr\} ^{\frac{1}{p}}< B \biggl(\frac{1}{q},\lambda - \frac{1}{q} \biggr) \biggl( \int _{0}^{\infty } f^{p}(x)\,dx \biggr)^{\frac{1}{p}}, $$

31 $$ \Biggl\{ \int _{0}^{\infty } \Biggl[ \sum _{n = 1}^{\infty } \frac{a_{n}}{(1 + xn)^{\lambda }} \Biggr]^{q} \,dx \Biggr\} ^{\frac{1}{q}}< B \biggl(\frac{1}{q},\lambda - \frac{1}{q} \biggr) \Biggl(\sum_{n = 1}^{\infty } n^{q - 2}a_{n}^{q} \Biggr)^{\frac{1}{q}}. $$

(iii) For $\lambda = 1$, $\sigma = \frac{1}{p}$, $\mu = \frac{1}{q}$ in (23), (24) and (25), we have the following equivalent inequalities with the homogeneous kernel and the best possible constant factor $\frac{\pi }{\sin (\frac{\pi }{p})}$:

32 $$ \int _{0}^{\infty } \sum_{n = 1}^{\infty } \frac{a_{n}}{x + n} f(x)\,dx< \frac{\pi }{\sin (\frac{\pi }{p})} \biggl( \int _{0}^{\infty } f^{p}(x)\,dx \biggr)^{\frac{1}{p}} \Biggl(\sum_{n = 1}^{\infty } a_{n}^{q} \Biggr)^{\frac{1}{q}}, $$

33 $$ \Biggl[\sum_{n = 1}^{\infty } \biggl( \int _{0}^{\infty } \frac{f(x)}{x + n}\,dx \biggr)^{p} \Biggr]^{\frac{1}{p}}< \frac{\pi }{\sin (\frac{\pi }{p})} \biggl( \int _{0}^{\infty } f^{p}(x)\,dx \biggr)^{\frac{1}{p}}, $$

34 $$ \Biggl[ \int _{0}^{\infty } \Biggl( \sum _{n = 1}^{\infty } \frac{a_{n}}{x + n} \Biggr)^{q} \,dx \Biggr]^{\frac{1}{q}}< \frac{\pi }{\sin (\frac{\pi }{p})} \Biggl(\sum _{n = 1}^{\infty } a_{n}^{q} \Biggr)^{\frac{1}{q}}. $$

(iv) For $\lambda = 1$, $\sigma = \frac{1}{q}$, $\mu = \frac{1}{p}$ in (23), (24) and (25), we have the following equivalent inequalities with the best possible constant factor $\frac{\pi }{\sin (\frac{\pi }{p})}$:

35 $$ \int _{0}^{\infty } \sum_{n = 1}^{\infty } \frac{a_{n}}{x + n} f(x)\,dx< \frac{\pi }{\sin (\frac{\pi }{p})} \biggl( \int _{0}^{\infty } x^{p - 2}f^{p}(x)\,dx \biggr)^{\frac{1}{p}} \Biggl(\sum_{n = 1}^{\infty } n^{q - 2}a_{n}^{q} \Biggr)^{\frac{1}{q}}, $$

36 $$ \Biggl[\sum_{n = 1}^{\infty } n^{p - 2} \biggl( \int _{0}^{\infty } \frac{f(x)}{x + n}\,dx \biggr)^{p} \Biggr]^{\frac{1}{p}}< \frac{\pi }{\sin (\frac{\pi }{p})} \biggl( \int _{0}^{\infty } x^{p - 2}f^{p}(x)\,dx \biggr)^{\frac{1}{p}}, $$

37 $$ \Biggl[ \int _{0}^{\infty } x^{q - 2} \Biggl( \sum _{n = 1}^{\infty } \frac{a_{n}}{x + n} \Biggr)^{q} \,dx \Biggr]^{\frac{1}{q}}< \frac{\pi }{\sin (\frac{\pi }{p})} \Biggl(\sum _{n = 1}^{\infty } n^{q - 2}a_{n}^{q} \Biggr)^{\frac{1}{q}}. $$

## Conclusions

In this paper, by using the weight functions and the idea of introducing parameters, a half-discrete Hilbert inequality with the nonhomogeneous kernel and its equivalent form are given in Theorem 1. The equivalent statements of the constant factor being best possible related to parameters, and some particular cases are considered in Theorem 2 and Remark 1. The cases of homogeneous kernel are deduced in Corollary 1 and Corollary 2. The lemmas and theorems provide an extensive account of this type of inequalities.
